# Eplerenone for early cardiomyopathy in Duchenne muscular dystrophy: results of a two-year open-label extension trial

**DOI:** 10.1186/s13023-017-0590-8

**Published:** 2017-02-20

**Authors:** Subha V. Raman, Kan N. Hor, Wojciech Mazur, Xin He, John T. Kissel, Suzanne Smart, Beth McCarthy, Sharon L. Roble, Linda H. Cripe

**Affiliations:** 1 Ohio State University, 473 W. 12th Ave, Suite 200, Columbus, OH 43210 USA; 2 Nationwide Children’s Hospital, Columbus, OH USA; 3 The Christ Hospital Heart and Vascular Center, Cincinnati, OH USA; 4 Department of Epidemiology and Biostatistics, University of Maryland, College Park, Maryland, USA; 5 The Ohio State University Department of Neurology, Columbus, OH USA

**Keywords:** Cardiomyopathy, Eplerenone, Mineralocorticoid receptor antagonist, Duchenne, Muscular dystrophy

## Abstract

**Background:**

Cardiomyopathy is a leading cause of morbidity and mortality in boys with Duchenne muscular dystrophy (DMD). We recently showed in a 12-month double-blind randomized controlled trial that adding eplerenone to background medical therapy was cardioprotective in this population. The objective of this study was to evaluate the safety and efficacy of longer-term eplerenone therapy in boys with DMD.

**Results:**

Eleven subjects (phase 1 baseline median [range] age: 13 [7 – 25] years) from the original 12-month trial at a single participating center were enrolled. Importantly, those who entered the extension study who had been on eplerenone previously were significantly older than those who had originally been on placebo (median age 10.5 vs. 18.0 years, *p* = 0.045). During an additional 24-month open-label extension study, all boys received eplerenone 25 mg orally once daily to treat preclinical DMD cardiomyopathy, defined as evident myocardial damage by late gadolinium enhancement cardiac magnetic resonance (LGE) with preserved ejection fraction (EF). The threshold for potassium level, the primary safety measure, was not exceeded in any non-hemolyzed blood sample. Over 24 months, left ventricular (LV) systolic strain, a more sensitive marker whose more negative values indicate greater contractility significantly improved (median change -4.4%, IQR -5.8 to -0.9%) in younger subjects whereas older subjects’ strain remained stable without significant worsening or improvement (median change 0.2%, IQR -1.1 to 4.3%). EF and extent of myocardial damage by LGE remained stable in both groups over 2 years.

**Conclusions:**

Eplerenone offers effective and safe cardioprotection for boys with DMD, particularly when started at a younger age. Eplerenone is a useful clinical therapeutic option, particularly if treatment is initiated earlier in life when cardiac damage is minimal.

**Trial registration:**

http://ClinicalTrials.gov identifier NCT01521546. Registered 26 January 2012.

## Background

Duchenne muscular dystrophy (DMD) affects approximately one of every 3,300 male births worldwide [1], and is caused by mutations in the gene encoding for the myocyte structural protein dystrophin [2]. Loss of dystrophin leads to inexorable damage to skeletal and cardiac muscle, with cardiomyopathy increasingly recognized as a major cause of death [3]. Animal models and human data indicate that myocardial damage occurs well before functional decline such as drop in left ventricular (LV) ejection fraction (EF) is apparent [4], endorsing a strategy of treatment while EF is preserved. A more sensitive measure of cardiac function, LV systolic strain, has high reproducibility when measured by cardiac magnetic resonance [5] – a critical feature when testing hypotheses in patients with rare diseases to support efficient sample size reductions [6] – and is abnormal in even the youngest boys with DMD well before EF drops [7].

The availability of early, sensitive markers of myocardial damage and dysfunction warrants effective therapies that can be readily instituted. While considerable advances are being made in gene therapy [8], there remains a need for immediately-available cardioprotective agents that can serve DMD patients across mutations and with more advanced skeletal myopathy. In a recently-published randomized, double-blind 12-month clinical trial, we demonstrated that adding the available mineralocorticoid receptor antagonist drug eplerenone to background therapy was superior to placebo in boys with DMD and preserved EF in attenuating decline of cardiac function [9]. In this open-label extension study, we tested the hypothesis that eplerenone would have a durable benefit on cardiac function preservation.

## Methods

### Study design, patient selection, and treatment

Enrollment in this open-label extension study (NCT01521546) was offered to subjects completing their participation in a 12 month placebo-controlled, randomized, double-blind trial of eplerenone, noting that 2 of the original study centers (University of California Los Angeles and Cincinnati Children’s Hospital Medical Center) did not participate in the extension phase due to subject preference, other trials, or limited resources [9]. Twenty individuals age 7 years or older completed the study from the Ohio State University (OSU) and Nationwide Children’s Hospital (NCH) with a diagnosis of DMD by mutation analysis, and were eligible for enrollment in the extension phase. Included in the original study were patients with evident myocardial damage by late gadolinium enhancement CMR and preserved left ventricular (LV) systolic dysfunction, defined by a CMR LV ejection fraction of ≥ 45%. Background therapy at the time of enrollment included angiotensin converting enzyme inhibitor (ACEI) or angiotensin receptor blocker (ARB) in all subjects, and none were previously on eplerenone or spironolactone. Additional detail regarding inclusion and exclusion criteria for the original study has been described [9]. The extension study and associated data safety monitoring plan were approved after institutional review board review that required additional i) written informed consent for subjects ≥18 years, ii) participant assent plus parent or guardian permission for subjects age 14 to 17 years or iii) parental permission alone for participants younger than 14 years of age beyond what was provided for participation in the original study. Study data were electronically captured and managed in accordance with all regulatory requirements [10]. Treatment during the extension phase consisted of eplerenone 25 mg, one tablet by mouth daily.

### Safety and efficacy assessments

All subjects had potassium meticulously monitored during the initial 12 month study. Serum potassium measurements were measured 1 month after entry in the extension phase and then annually (i.e. at 24 and 36 months after baseline) with additional measurements on a case-by-case basis as dictated by each subject’s clinical team. Telephone follow-up calls with drug diary review were conducted at 3, 6 and 9 months of each year in addition to annual subjects’ clinic visits that included cardiology and neurology appointments.

Repeat CMR examination was performed annually for up to 2 additional years using the identical protocol and 3 Tesla scanner (Siemens Verio, Erlangen, Germany) as in the original study: 1) long axis and short-axis cine imaging using steady-state free precession spanning the LV to compute volumes and EF, mid-short-axis tagged cine for computation of LV strain, and short-axis LGE covering the LV for myocardial damage assessment. LGE images used inversion-recovery gradient echo acquisitions 12–15 min after intravenous administration of 0.2 mmol/kg gadopentetate dimeglumine in the identical long axis and short axis planes as cine imaging, with inversion time optimized to null normal myocardium. As in the original study, efficacy was primarily assessed by change in left ventricular circumferential strain with secondary outcome measures of change in LV volumes, ejection fraction, and myocardial extent of late gadolinium enhancement. Adverse events and any admissions to hospital because of heart failure, documented arrhythmias, death, or hyperkalemia (potassium concentration ≥5.5 mmol/L) were recorded via telephone interviews and at clinic visits.

### Statistical analysis

The median values of the 12-month change (from 12 to 24 months) and 24-month change (from 12 to 36 months) in strain, LV EF and LGE during this open-label extension phase (from 12 to 36 months) were compared between the eplerenone and placebo groups, using two-sample Wilcoxon rank-sum tests.

## Results

### Patient disposition and demographic characteristics

Eleven of the original 20 male subjects who completed the study from OSU/NCH elected to participate in the open-label extension phase; 3 declined participation due to unwillingness to undergo additional MRIs, and 6 declined participation due to difficulty with additional travel. Median age at initial study entry was 13 years, range 7 to 25 years; 6 participants were on placebo and 5 on eplerenone during the initial double-blind year of the trial, with those originally on placebo significantly younger than those originally on eplerenone (median age 10.5 vs. 18.0 years, *p* = 0.045). Background ACEI or ARB therapy was mandated prior to enrollment in the original study, and continued in all participants (10 taking an ACEI and 1 taking an ARB) throughout the extension phase. Five subjects were on a beta-blocker during the extension study. Types and dosages of background medications included: lisinopril (2.5 – 5 mg qd), enalapril (5 mg bid), losartan (25 mg qd), metoprolol succinate (25 mg qd), carvedilol (3.125 mg bid). Dose changes in background therapy over the course of the extension study included one subject age 18 years originally assigned to eplerenone whose enalapril dose was increased by 5 mg and two subjects ages 9 and 15 years originally assigned placebo whose lisinopril doses were increased by 2.5 mg.

### Safety profile

**Fig. 1:**
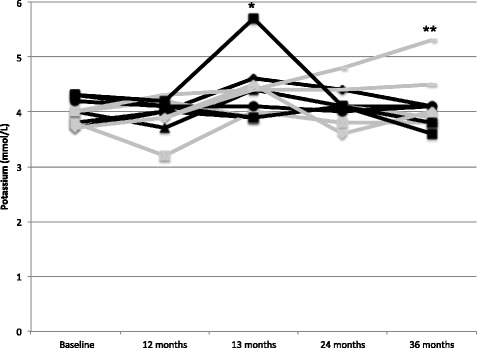
Safety. Serial potassium values from baseline through the end of the 36-month open label extension period are shown; *black lines* indicate younger subjects who were on placebo and *gray lines* were older subjects on eplerenone during the initial 12-month double blind RCT. One result in a subject initially on placebo was 5.7 mmol/L (*) at 13 months (1 month after starting open-label eplerenone); this was repeated and found to be 3.7 mmol/L. Potassium level in a hemolyzed sample at the 36 month visit was 5.3 mmol/L (**)

Potassium levels at entry into the extension trial averaged 4.0 ± 0.3 mmol/L; a plot of serial potassium values in the 11 subjects is shown in Fig. 1. Median of change in potassium level over 36 months was -0.2 mmol/L in the 6 subjects initially on placebo and 0.2 mmol/L in the 5 who were on eplerenone from the beginning. One subject initially on placebo during the RCT had a 13-month (1 month after starting open-label eplerenone) potassium level of 5.7 mmol/L with concern regarding hemolysis; this prompted temporary cessation of study medication that was resumed after repeat measurement showed a level of 3.7 mmol/L. Another subject reported discolored urine that resolved after discontinuation of background idebenone therapy. There were no episodes of hospitalization, arrhythmia, heart failure, or death.

### Follow-up efficacy data

**Fig. 2:**
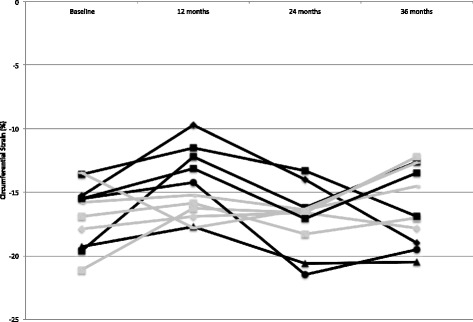
Efficacy. Serial left ventricular circumferential strain values from baseline through the end of the 36-month open label extension period are shown; *black lines* indicate younger subjects who were on placebo and *gray lines* were older subjects on eplerenone during the initial 12-month double blind RCT

After an initial 12 months on placebo, younger boys realized significant improvement in LV strain (i.e. more negative) over the first 12 months of open-label extension therapy (median change -4.0%, IQR -4.3 to -2.9%) that persisted over the 24-month extension period (median change -4.4%, IQR -5.8 to -0.9%). The older boys who were on eplerenone during the initial double-blind 12 months maintained strain over the subsequent 12-month open label extension period (median change -0.3%, IQR -1.2 to 0.3%). This stability was retained over the 24-month extension period (median change 0.2%, IQR -1.1 to 4.3%). The strain improvement realized in the younger boys was significantly better compared to the stability or lack of continued improvement in the older boys (*p* = 0.0106 for first 12-months of extension and *p* = 0.0446 for months 13-24 of extension; Fig. 2).

LV EF was relatively stable over an extension period of 12 months (younger boys initially on placebo: median change -1.9%, IQR -2.3 to 1.5%; older boys initially on eplerenone: median -1.2%, IQR -5.0 to 2.2%; *p* = 0.7150) and 24 months (younger boys initially on placebo: median change 2.7%, IQR 1.4 to 3.8%; older boys initially on eplerenone: median change -1.2%, IQR -7.9 to 1.0%; *p* = 0.0541). This EF stability reflected a median change over 24 months in end-diastolic volume of 16.7 mL (IQR 10.3 to 29.5 mL) in younger boys initially on placebo vs. 4.2 (IQR 2.8 to 13.0 mL) in older boys initially on eplerenone (*p* = 0.1003); the median change in end-systolic volumes in the two groups were 5.3 mL (IQR 0.6 to 17.5 mL) and 4.7 mL (IQR 4.2 to 4.8 mL) (*p* > 0.9999), respectively.

Similar patterns of change were observed in LGE over the extension period in 12 months (younger boys initially on placebo: median change -0.5 segments, IQR -1.0 to 1.0 segments; older boys initially on eplerenone: median -1.0 segment, IQR -1.0 to -1.0 segments; *p* = 0.6304) and in 24 months (younger boys initially on placebo: median 0 segments, IQR 0 to 2.0 segments; older boys initially on eplerenone: median 0 segments, IQR -2.0 to 1.0 segments; *p* = 0.5637).

## Discussion

In this 24-month open label extension study, we found a striking benefit on cardiac function in young boys with DMD. While the sensitive marker of subclinical cardiac dysfunction LV strain was also stabilized in older boys, the benefit was attenuated compared to that seen with eplerenone therapy at a younger age though still with stabilization in strain compared to the previously reported decline without therapy [11]. The older subjects happened to be those who had already received eplerenone during the prior 12 month double-blind trial, and may have already realized the largest magnitude of benefit by adding MRA therapy to background ACEI or ARB. Importantly, no subjects experienced an adverse potassium level, the primary safety concern that has emerged from MRA trials in other populations. These results are the first clinical data of MRA therapy supporting earlier vs. later institution of treatment, consistent with results from the DMD mouse that endorsed greater efficacy with earlier therapy [12]. Our findings expand the literature endorsing safety of eplerenone for pediatric patients [9, 13], and amplify a recent recommendation to consider spironolactone for patients with heart failure and preserved ejection fraction amidst heterogeneous trial data [14].

In using an LVEF cutoff of ≥45% for baseline enrollment, we sought to enroll subjects with preserved EF; this may not equate to a ‘normal’ CMR EF per reference values for healthy children and adults (Sarikouch 2010, Kawel-Boehm 2015). Patients with heart failure with preserved EF (HFpEF) have very distinct responses to conventional medical therapies compared to those with heart failure and reduced EF (HFrEF) (Yancy 2013), and the early DMD cardiomyopathy phenotype is more consistent with preserved EF. Thus, this cutoff allows our trial results to align with the limited but growing body of evidence relevant to treatment decision-making for boys with early DMD cardiomyopathy.

The parent RCT required evident myocardial injury as seen by LGE-CMR for enrollment; thus, we cannot speculate as to the potential efficacy in boys who are still LGE-negative. Future trials targeting DMD boys at the time of diagnosis may be warranted. We also recognize the exciting potential for emerging genetic therapies to treat the underlying defect. While such therapies continue to expand beyond the small range of target mutations for which they are presently effective and require further optimization to deliver myocardial benefit, MRA therapy may offer a safe therapeutic approach to cardioprotection. The small sample and effect sizes of this study of a rare disease for which there are a number of active enrolling trials of alternate therapies is a limitation, as is heterogeneity in background medication use, though the results suggest benefit without significant risk.

## Conclusions

In conclusion, younger boys treated with eplerenone realize a significant improvement in LV systolic function over 24 months of therapy. Older boys whose therapy was continued for an additional 24 months showed no significant worsening in LV systolic function with this treatment. Early MRA therapy in boys with DMD warrants consideration to achieve the greatest likelihood of cardiac benefit.

## Abbreviations

ACEI: Angiotensin converting enzyme inhibitor
ARB: Angiotensin receptor blocker
CMR: Cardiovascular magnetic resonance
DMD: Duchenne muscular dystrophy
EF: Ejection fraction
IQR: Interquartile range
LGE: Late gadolinium enhancement
LV: Left ventricle
MRA: Mineralocorticoid receptor antagonist
RCT: Randomized controlled trial
